# Private and well drinking water are reservoirs for antimicrobial resistant bacteria

**DOI:** 10.1038/s44259-024-00024-9

**Published:** 2024-03-18

**Authors:** Marwa Alawi, Cian Smyth, David Drissner, Anna Zimmerer, Denise Leupold, Daria Müller, Thi Thuy Do, Trinidad Velasco-Torrijos, Fiona Walsh

**Affiliations:** 1 Department of Biology, Maynooth University, Maynooth, County Kildare Ireland; 2 Kathleen Lonsdale Institute for Human Health Research, Maynooth University, Maynooth, County Kildare Ireland; 3 Department of Life Sciences, Albstadt-Sigmaringen University, 72488 Sigmaringen, Germany; 4 Department of Agriculture, Food and the Marine, Celbridge, Kildare, Ireland; 5 Department of Chemistry, Maynooth University, Maynooth, County Kildare Ireland

**Keywords:** Environmental sciences, Water microbiology

## Abstract

Water quality testing does not recognise antimicrobial resistance (AMR) and is often limited to indicators of faecal contamination *Escherichia coli* and *Enterococcus* species. In Europe, data on AMR in drinking water is scarce. In Ireland, as in many countries, household drinking water is supplied via mains or via private wells or water schemes. Using citizen science, we identified Irish private drinking water supplies as reservoirs of antimicrobial resistant bacteria (ARB). Gram-negative (*n* = 464) and Gram-positive (*n* = 72) bacteria were isolated. We identified instances of potentially opportunistic ARB such as *Enterobacter cloacae, Acinetobacter baumannii* and *Enterococcus* species. We report reservoirs of multidrug resistance in *Enterococcus casseliflavus, E. cloacae, E. coli, Stenotrophomonas maltophilia*, and *Serratia rubidaea*. We also identified linezolid-resistant *Enterococcus* in Irish drinking water. Linezolid is a last-resort antibiotic used to treat vancomycin-resistant *Enterococcus* sp. Additionally, we identified mobile AMR in three water samples, two of which were carried on IncF group, one on IncQ and five on Col-like plasmids. Our work suggests that private drinking water is a potential sink and source of AMR pathogens. This highlights a value of drinking water surveillance in a One Health framework as the surveillance would provide information regarding the movement and persistence of ARB and ARGs that are able to survive in drinking water and subsequently have the opportunity to be mobilised through humans; linking the environment to the human and potentially threatening human health.

## Introduction

The World Health Organisation sets out minimum international guidelines for mitigating faecal contaminants in drinking water^1^. Drinking water becomes contaminated with faecal coliforms due to anthropogenic activities such as manure spreading and leakage of wastewater treatment systems^2,3^. While the current water quality guidelines aim to reduce instances of faecal contamination, they fail to consider the potential of water to be a reservoir for antimicrobial resistance genes (ARGs) conferring resistance against clinically relevant antimicrobials. Antimicrobial resistance (AMR) is a global epidemic that is recognised by the World Health Organisation as one of the top ten public health issues facing the human population^4^ and makes up a key component of the One Health approach to improving public health outcomes^5^. Horizontal gene transfer (HGT) of mobile resistance elements has been documented as a leading cause for the dissemination and persistence of ARGs^6^. In recent years, studies have revealed the presence of antimicrobial resistant bacteria (ARB) in drinking water supplies globally^7–13^. The emergence of ARB and ARGs in drinking water raises concerns regarding the transmission of AMR from the environment to the human and the potential impact on human health. In the human gut, HGT is a common occurrence^14^. The potential for bacteria to acquire ARGs via HGT in the gut following consumption of water contaminated with ARB has been explored in mice^15^ however, the interlink between consumption of ARGs through drinking water and the impact this has on the human gut microbiome is yet to be explored. If this potential is realised in the human gut, this could possibly lead to gastrointestinal illnesses^16^ and even bloodstream infections that are more challenging to treat^17^.

The aim of this work was to employ citizen science to isolate and identify bacterial reservoirs of antimicrobial resistance in private sources of drinking water in Ireland and to elucidate the role of mobile AMR in the drinking water resistomes.

## Results

### Water samples and household background

The water sampling and citizen science project occurred during the COVID pandemic, where tight restrictions on movement were in place across Ireland. We collected data regarding the sampling locations and the use of antimicrobials in each household by means of a written questionnaire (Supplementary Questionnaire 1). Questionnaires from all volunteers (*n* = 49 households) situated across Ireland were received. Several households (*n* = 19) relied on privately sourced drinking water. Of those, 18 reported owning a private well, one reported sourcing water from a spring. Additionally, 30 households relied on Group Water Schemes (GWS). The GWS supplied drinking water via springs (*n* = 16, 53%) or wells (*n* = 5, 17%). The remainder of households supplied by GWS (*n* = 9, 30%) did not indicate the source of their drinking water. Of the households that reported wells as their source of drinking water (*n* = 23), the majority reported having a bored (*n* = 10, 43%) or drilled well (*n* = 8, 35%) and, dug wells (*n* = 3, 13%) were the least common. One household reported that their well was both dug and drilled. Two households did not report how the wells were constructed. A significant number of households did not use filtration (*n* = 44, 90%) nor treatment systems (*n* = 43, 88%) to reduce microbial contamination in their drinking water. Only six (12%) households reported the use of chlorination, five of which were supplied water from the same GWS, the other is supplied by a different GWS. Of these households, one also reported the use of ultraviolet (UV) treatment. The GWS and private wells were constructed between 1973 and 2019. The depth of the wells ranged between 1.3 m–160 m.

Overall, the majority (*n* = 33, 67%) of households reported living on or within 5 km of farmland. Although not asked to specify, one household reported the presence of sheep and cattle on the household farm (Household 6) and one household reported having horses on the farm and the presence of a cattle farm within 1 km of residence (Household 44). Most households (*n* = 31, 63%) considered their surroundings to be remote rural or rural community. A smaller number (*n* = 18, 37%) reported proximity to surface waters such as rivers and lakes. Others reported living near quarries (*n* = 12, 24%), landfills (*n* = 4, 8%) and/or manufacturing and processing facilities (*n* = 4, 8%). Households living on farms (*n* = 12, 24%) did not use antibiotics on their farm animals at any stage since the arrival of the animals to the farms, whilst over 59% (*n* = 29) of households reported use of antibiotics in the past 10 years to treat human infections. The beta-lactam antibiotics (penicillin, amoxicillin-clavulanate) were the most frequently reported (*n* = 20, 41%). Cephalosporins and other antibiotics were less common (*n* = 2, 4%). A small number of households (*n* = 8, 16%) were either unsure or did not indicate the type of antibiotics they have consumed.

### Correlation analysis

We assessed for correlation between the reliance on domestic wastewater systems the detection of ARB in drinking water samples. We identified a moderate positive correlation between the use of septic tanks and the prevalence of antimicrobial resistant bacteria (Spearman’s r_S_ value + 0.649, *α* = 0.001). Additionally, correlation analysis for households that reported the use of antimicrobials revealed Spearman’s r_S_ value of + 0.716 (*α* = 0.002), suggesting a positive correlation between the use of antimicrobials and the presence of bacteria of clinical relevance. These households were more likely to harbour potentially pathogenic species (e.g. *E. faecium, E. cloacae, S. maltophilia, E. coli*).

### Isolation and identification of bacterial reservoirs of AMR

We isolated bacteria on selective agars with and without antibiotics and used MALDI-TOF-MS and 16 S rRNA sequencing to speciate the isolates which were subsequently tested against a variety of antimicrobials. Of 49 household samples retrieved, 21 arrived later than 24 h after sampling. We isolated bacteria from all but one sample arriving to our laboratory within 24 h of sample collection (*n* = 21) (Supplementary Table 1). As this work focuses on AMR bacteria rather than quantification of faecal bacteria, we deviated from ISO quality testing standards and processed all samples, including those arriving beyond the recommended 24 h mark. In some instances, we were able to isolate bacteria from samples arriving beyond the 24 h period (*n* = 6) but we were only able to isolate bacteria from one household where the water sample arrived 96-h after collection. The longest time between collection and processing was 96 h.

**Fig. 1:**
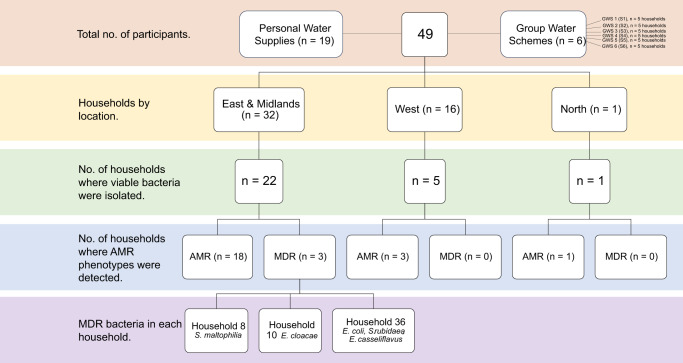
Flow chart summary of key outputs of sampling and analysis. Household and Group Water Scheme numbers are confidential identifiers. The households are classified based on private ownership of the water supply or dependence on Group Water Schemes (S1–S6). The geographical breakdown of households is focused on the East & Midlands, the West or Northern Ireland. For each region, the number of households harbouring viable bacteria is indicated. These bacteria are grouped based on phenotype (AMR or MDR). The households and species which harboured MDR isolates are shown. GWS: Group Water Scheme, AMR: Antimicrobial-Resistant, MDR: Multidrug-Resistant.

Overall, 536 isolates constituting 464 Gram-negative and 72 Gram-positive bacteria were isolated from 28 of 49 household samples. Bacterial isolates were not detected in samples from 21 households, 20 of which were those that were delayed (>24 h) arriving to the laboratory (Supplementary Table 1) and thus it is possible that a loss of viability occurred in this time period. Bacteria resistant to at least one class of antimicrobial were detected in 22 of 28 (78.6%) households (Fig. 1).

In total, 244 of 536 isolates (45.5%) were resistant to one or more of the antimicrobial agents tested. We identified a range of bacteria acting as reservoirs for antimicrobial resistance against a broad-range of antimicrobial classes such as the beta-lactams, cephalosporins and fluoroquinolones amongst others (Table 1). Ampicillin-resistance was found predominantly in species which are known to produce intrinsic AmpC beta-lactamases such as *Citrobacter* sp. And *Buttiauxella* sp. AmpC producers made up 68.5% (*n* = 367) of total isolated bacteria. Acquired ampicillin-resistance was detected in *E. faecium* and *E. coli* isolated from Household 36. Separately, five households from the same Group Water Scheme (S6) were also reservoirs for ampicillin-resistant *E. faecium*.

**Table 1:** Summary of bacterial reservoirs of AMR isolated from household drinking water

Antimicrobial Agent	Species	No. of households	Total no. isolates
**Ampicillin**	*Escherichia coli*	1	19
	*Enterococcus faecium*	6	23
**Cefotaxime**	*Acinetobacter baumannii*	1	1
	*Citrobacter gillenii*	2	2
	*Enterobacter cloacae*	4	5
	*Enterobacter hormaechei*	1	1
	*Hafnia alvei*	1	9
	*Raoultella planticola*	1	1
	*Serratia fonticola*	1	1
	*Serratia rubidaea*	1	4
**Ceftazidime**	*Acinetobacter schindleri*	1	1
	*Hafnia alvei*	1	3
	*Raoultella planticola*	1	2
	*Stenotrophomonas maltophilia*	5	57
	*Serratia rubidaea*	1	1
**Ertapenem**	*Hafnia alvei*	1	2
**Trimethoprim**	*Brevundimonas aurantiaca*	1	1
	*Enterobacter cloacae*	1	7
	*Hafnia alvei*	1	2
	*Proteus hauseri*	1	2
	*Serratia rubidaea*	1	2
	*Yersinia massiliensis*	1	1
**Chloramphenicol**	*Enterobacter cloacae*	2	3
	*Escherichia coli*	1	22
	*Stenotrophomonas maltophilia*	3	47
**Tetracycline**	*Enterococcus casseliflavus*	1	2
	*Enterobacter cloacae*	1	2
	*Escherichia coli*	1	17
	*Enterococcus durans*	1	13
	*Enterococcus faecium*	3	10
	*Enterococcus hirae*	1	6
	*Serratia rubidaea*	1	4
**Ciprofloxacin**	*Acinetobacter baumannii*	1	1
	*Brevundimonas aurantiaca*	1	1
	*Bacillus megaterium*	1	1
	*Enterococcus casseliflavus*	1	2
	*Enterococcus faecium*	1	1
	*Serratia fonticola*	1	1
	*Serratia rubidaea*	1	2
**Levofloxacin**	*Stenotrophomonas maltophilia*	1	2
**Linezolid**	*Enterococcus casseliflavus*	1	1
	*Enterococcus durans*	1	1
	*Enterococcus faecium*	1	1
**Erythromycin**	*Enterococcus faecium*	1	4

Carbapenem-resistance was predominantly identified in known carbapenemase producing species *S. maltophilia*, which made up 8.4% (*n* = 45) of total isolated bacteria. Only two isolates of *H. alvei* from the same household were resistant to ertapenem. No other carbapenem-resistance was identified. Cephalosporin-resistance was identified in ten species of bacteria isolated from twelve unique households. Cefotaxime-resistance was more frequently identified compared to ceftazidime.

*Enterococcus* sp. from two unrelated households were susceptible to vancomycin but were resistant to the last-resort antimicrobial, linezolid. These species were *E. durans and E. faecium*. Additionally, linezolid-resistance was detected in *E. casseliflavus* which is known to carry a chromosomal *vanC* gene allowing the expression of low-level vancomycin-resistance^18^.

As our methods did not follow the ISO standards for faecal contamination detection using *E. coli* and *Enterococcus* sp. the presence of *E. coli* and *Enterococcus* sp. may be below the limit of detection of the ISO tests due to the variation in our methods. Therefore, we cannot state that faecal contamination has occurred. *Escherichia coli* and *Enterococcus* sp were amongst the bacteria identified in 10% (*n* = 5) and 20% (*n* = 10) of households, respectively. 60% of all *E. coli* (*n* = 24 of 40) and 73% of all *Enterococcus* (53 of 72) isolated were resistant to at least one class of antimicrobials.

**Fig. 2:**
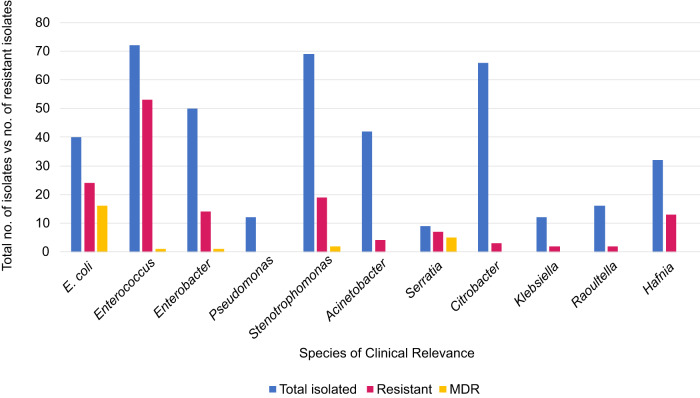
AMR species of clinical relevance identified in private drinking water. The bar chart shows the different species of clinical relevance and the numbers which were identified as being antimicrobial resistant. The blue denotes the total number of isolates from each species of relevance, red signifies the number of isolates identified as resistant to at least one class of antimicrobial and yellow indicates resistance to at minimum one class of antimicrobials. MDR: Multidrug-Resistant.

We also identified AMR species of clinical relevance (Fig. 2) including potentially opportunistic pathogens. Antimicrobial-resistant *Enterobacter* sp. made up 28% (14 of 50) of isolated *Enterobacter* sp., thirteen of which were *E. cloacae* and one *E. hormaechei*. Antimicrobial resistant *S. maltophilia* made up 27.5% (19 of 69) of isolated *S. maltophilia* and AMR *Acinetobacter* sp. made up 9.5% (4 of 42) of isolated *Acinetobacter* sp. Three were *A. baumannii* and one *A. schindleri*. Whilst a large number of *Citrobacter* sp. were isolated (*n* = 66) including twenty *C. freundii* (30%) isolates, AMR was only identified in *C. gillenii* (*n* = 3, 4.5%). No AMR *Pseudomonas* sp. were identified.

**Fig. 3:**
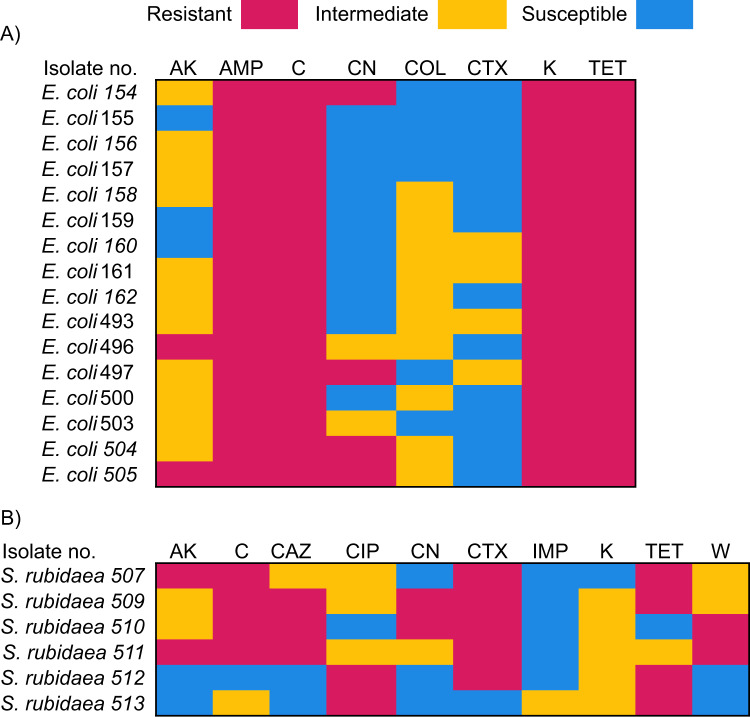
Resistance patterns of antimicrobial resistant bacteria isolated from drinking water belonging to household 36. The resistance patterns for multidrug-resistant (**a**) *Escherichia coli* and (**b**) antimicrobial resistant *Serratia rubideae* shows similarity in resistance patterns against phenicols, aminoglycosides and tetracycline. Blue represents susceptibility, yellow signifies intermediate resistance and red denotes resistance of the isolate to the antimicrobial. The numbers assigned are arbitrary identifiers. *AK* amikacin, *AMP* ampicillin, *C* chloramphenicol, *CN* gentamicin, *COL* colistin, *CTX* cefotaxime, *K* kanamycin, *TET* tetracycline, *CAZ* ceftazidime, *CIP* ciprofloxacin, *IMP* imipenem, *W* trimethoprim.

Three households from the East & Midlands (Household 8, 10 and 36) were reservoirs for MDR bacteria. The MDR bacteria isolated included two *S. maltophilia* (Household 8) isolates which were resistant to ceftazidime, chloramphenicol, and levofloxacin. One isolate of *E. cloacae* (Household 11) was resistant to cefotaxime, tetracycline, and ciprofloxacin. From Household 36, sixteen isolates of *E. coli* and five isolates of *S. rubidaea* were resistant to multiple combinations of antimicrobial classes (Fig. 3) including the tetracyclines and phenicols. One isolate of *E. casseliflavus* (Household 36) was resistant to linezolid, tetracycline, and chloramphenicol. These households have a few things in common: they all report living in a remote rural area, their water supply is a well that is 20+ years old and between 15–30 m in depth, all three depend on a domestic wastewater treatment system and none of them report treating their water against microbial contamination. While these characteristics were not unique to these three households, the aforementioned are a few risk factors that may have contributed to the presence of MDR bacteria.

Overall, the identification of bacteria of clinical relevance (*E. coli, Enterococcus* sp*., Enterobacter* sp*., S. rubidaea, S. maltophilia*) in drinking water as reservoirs for MDR is of most concern.

### Mechanisms of antimicrobial resistance

To identify the mechanism of reduced susceptibility towards the fluoroquinolone ciprofloxacin in 20 isolates, we screened for PMQR genes and mutations associated with the QRDR regions. We did not identify any PMQR genes. Mutations in QRDR were mainly identified in isolates exhibiting intermediate susceptibilities towards ciprofloxacin with two exceptions observed in ciprofloxacin-resistant *S. rubidaea* 512 and 513 (Table 2). In *E. coli* 505 the GyrA contained a mutation at amino acid position 87 (Asp→His). A mutation in the ParC of *H. alvei* 207 was identified at amino acid position 60 (Asn→Ser) and position 59 in *S. rubidaea* 507-513 (Ser→Thr). Mutations were identified in the GyrB of *S. rubidaea* 507-513 at amino acid position 439 (Arg→Lys) and *C. gillenii* 87 at amino acid positions 239 (Asn→Thr), 240 (Ile→Val), 287 (Ala→Ser) and 298 (Asp→Glu).

**Table 2:** Identification of mutations within the QRDR of bacterial isolates

Strain ID no.	Species	Ciprofloxacin resistance	Gene	Reference	Position	Mutation	Accession no. of susceptible comparator
**6**	*C. gillenii*	Intermediate	*gyrA*	-	-	-	QVEK01000004 QVEK01000004 CP033744
			*gyrB*	-	-	-	
			*parC*	-	-	-	
**87**	*C. gillenii*	Intermediate	*gyrA*	-	-	-	QVEK01000004 QVEK01000004 CP033744
			*gyrB*	Asn Ile Ala Asp	239 240 287 298	Thr Val Ser Glut	
			*parC*	-	-	-	
**144**	*E. cloacae*	Intermediate	*gyrA*	-	-	-	CP009756
			*gyrB*	-	-	-	
			*parC*	-	-	-	
**177**	*S. fonticola*	Intermediate	*gyrA*	-	-	-	LR134492
			*gyrB*	-	-	-	
			*parC*	-	-	-	
**206**	*H. alvei*	Intermediate	*gyrA*	-	-	-	NZ_CP050150
			*gyrB*	-	-	-	
			*parC*	-	-	-	
**207**	*H. alvei*	Intermediate	*gyrA*	-	-	-	NZ_CP050150
			*gyrB*	-	-	-	
			*parC*	Asp	60	Ser	
**226**	*C. gillenii*	Intermediate	*gyrA*	-	-	-	QVEK01000004 QVEK01000004 CP033744
			*gyrB*	-	-	-	
			*parC*	-	-	-	
**243**	*S. fonticola*	Resistant	*gyrA*	-	-	-	LR134492
			*gyrB*	-	-	-	
			*parC*	-	-	-	
**246**	*C. gillenii*	Intermediate	*gyrA*	-	-	-	QVEK01000004 QVEK01000004 CP033744
			*gyrB*	-	-	-	
			*parC*	-	-	-	
**296**	*E. asburiae*	Intermediate	*gyrA*	-	-	-	AP019632
			*gyrB*				
			*parC*	-	-	-	
The *gyrB* of *E. asburiae 296* could not be amplified using the set of primers listed in Supplementary Table 2, therefore was not assessed for mutations							
**302**	*E. cloacae*	Intermediate	*gyrA*	-	-	-	CP009756.1
			*gyrB*	-	-	-	
			*parC*	-	-	-	
**397**	*E. hormaechi*	Intermediate	*gyrA*	-	-	-	CP077392.1
			*gyrB*	-	-	-	
			*parC*	-	-	-	
**405**	*E. cloacae*	Intermediate	*gyrA*	-	-	-	CP009756
			*gyrB*	-	-	-	
			*parC*	-	-	-	
**484**	*A. baumannii*	Resistant	*gyrA*	-	-	-	CP043953
			*gyrB*	-	-	-	
			*parC*	-	-	-	
**505**	*E. coli*	Intermediate	*gyrA*	Asp	87	His	U00096
			*gyrB*	-	-	-	
			*parC*	-	-	-	
**507**	*S. rubidaea*	Intermediate	*gyrA*	-	-	-	NZ_CP065640
			*gyrB*	Arg	439	Lys	
			*parC*	Ser	59	Asn	
**509**	*S. rubidaea*	Intermediate	*gyrA*	-	-	-	NZ_CP065640
			*gyrB*	Arg	439	Lys	
			*parC*	Ser	59	Asn	
**511**	*S. rubidaea*	Intermediate	*gyrA*	-	-	-	NZ_CP065640
			*gyrB*	Arg	439	Lys	
			*parC*	Ser	59	Asn	
**512**	*S. rubidaea*	Resistant	*gyrA*	-	-	-	NZ_CP065643
			*gyrB*	Arg	439	Lys	
			*parC*	Ser	59	Asn	
**513**	*S. rubidaea*	Resistant	*gyrA*	-	-	-	NZ_CP065644
			*gyrB*	Arg	439	Lys	
			*parC*	Ser	59	Asn	

To identify carbapenemase activity, carbapenem-resistant *H. alvei* were exposed to EDTA in the presence of ertapenem to inhibit potential MBL activity. No MBL production in ertapenem-resistant *H. alvei* was detected. We also screened these isolates against a cohort of carbapenemase genes, none of which were detected by PCR. Additionally, we screened LRE isolates for mobile resistance genes *optrA, poxtA* and *cfr* but we did not detect any of these genes. Screening for mutations in the 23 S rRNA, L3, L4 and L22 ribosomal regions revealed a mutation in *E. durans* 62 in the 23 S rRNA sequence (A2227G) and in the L3-region at position 45 (Ser→Gly). No mutations were detected in the remaining LRE (Supplementary Table 5).

### Antimicrobial resistance plasmid isolation and characterisation

**Fig. 4:**
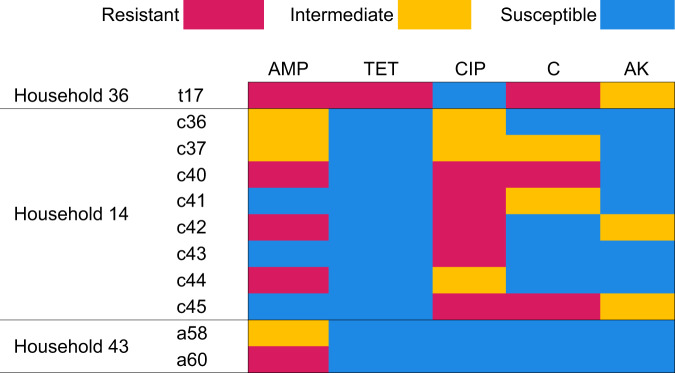
Antimicrobial susceptibility patterns of transconjugants. The resistance patterns of *E. coli* transconjugants from Households 36, 14 and 43 are shown where blue represents susceptibility, yellow signifies intermediate resistance and red denotes resistance of the transconjugant to the antimicrobial. The numbers assigned to each transconjugant are arbitrary identifiers. Letters next to the numbers represent the antimicrobial used to select the transconjugants: tetracycline (t), ciprofloxacin (c), amoxicillin (a). *AMP* ampicillin, *TET* tetracycline, *CIP* ciprofloxacin, *C* chloramphenicol, *AK* amikacin.

Exogenous plasmids were successfully transferred from three of the 28 investigated household drinking water samples to *E. coli* CV601, the recipient strain. No transconjugant growth was observed for the remaining household samples (*n* = 25). Antimicrobial susceptibility tests of transconjugants showed that transconjugants had acquired multiple antibiotic resistances (Fig. 4). Resistances against ampicillin, tetracycline, ciprofloxacin, and chloramphenicol were identified. Two instances of MDR transconjugants were observed (Transconjugants t17 and c40). Transconjugant t17 was selected in the presence of tetracycline and was resistant to ampicillin, tetracycline, and chloramphenicol. Transconjugant c40 was selected in the presence of ciprofloxacin and was resistant to ampicillin, ciprofloxacin, and chloramphenicol. These MDR transconjugants were obtained from households 14 and 36.

For household 36, the phenotype of Transconjugant t17 (ampicillin, tetracycline, chloramphenicol resistant) mirrored the phenotypes observed for the MDR *E. coli* and *S. rubidaea* (Fig. 3a, b) isolated from the same household which may be indicative of the host bacteria. However, the phenotypes of transconjugants c36-c45 from household 14 and transconjugants a58 and a60 from household 42 did not match to resistance phenotypes of isolates from the corresponding household.

We reduced the percentage identity to 50% (from the default 80%) when screening our plasmids. Doing so revealed one potential homologue of the *cfr*A gene in plasmid c36 which had 58.67% coverage and 99.52% identity match. All remaining ARGs and virulence factors identified were over 80% identical to those already deposited in the databases. Screening of plasmids identified conjugative machinery, replicon types, virulence factors and ARGs (Table 3). Plasmid sizes ranged between 33.6–118.8 kb. Transconjugant t17 carried multi-replicon plasmid with identity match 98.24% and 95.59% to IncFIB (Accession: JN233704) and IncFIC (Accession: AP001918) plasmids, respectively. The t17 plasmid was also 100% identical to IncQ (Accession: M28829), although the coverage was 66.46%. The t17 plasmid contained the beta-lactam resistance *bla*_TEM-1_ gene which confers the ampicillin-resistant phenotype observed. The *tetB* gene gave rise to a tetracycline-resistant phenotype. In addition, aminoglycoside-resistance genes of the *aphA*-variants were present. However, these genes did not confer resistance to gentamicin or amikacin. The *aphA(3’)-Ia* gene is known to confer resistance to kanamycin^19^. As the plasmids were expressed in an *E. coli* that was chromosomally resistant to kanamycin, we could not determine through disk testing if the *aphA*-variants were conferring resistance to kanamycin. The phenicol-resistance genes *cfrA, catI* and *floR* were present, which are responsible for the chloramphenicol-resistant phenotype. Sulfonamide-resistance gene, *sul2* was also present. Sequence analysis also identified virulence-associated *iroBCDEN* gene cluster, *iucABC* and *iutA*.

**Table 3:** Summary of plasmid analysis

ID	Bandage (visualisation)	Plasmid size (KB)	CARD (ABRicate)	resfinder (ABRicate)	VFDB (ABRicate)	Plasmid Finder (ABRicate)	No. of plasmid contigs (MOB-typer)	Replicon type (MOB-typer)	Relaxase type(s) (MOB-typer)	Predicted mobility (MOB-typer)	GenBank Accession numbers
**t17**		62.7	*aphA(3’)-Ia, aph(6)-Id, aph(3”)-Ib, sul2, TEM-1, tet(B), catI, floR*	*aph(3’)-Ia, aph(6)-Id, aph(3”)-Ib, sul2, bla*TEM-1B*, tet(B), catA1, floR*	*iroN, iroE, iroD, iroC, iroB, iucA, iucB, iucC, iucD, iutA, icsP/sopA*	IncFIB, IncFIC(FII)	2	IncFIA, IncFIB, IncFIC, IncQ1, rep_cluster_2232	MOBF, MOBF, MOBF, MOBP	conjugative	OR902841 OR902842
**c36**		118.8	*cfrA, TEM-116*	*cfr, bla*TEM-116	N/A	ColRNAI	2	rep_cluster_2350	-	non-mobilizable	OR902843 OR902844
**c37**		89.8	x	x	x	x	2	-	-	non-mobilizable	OR902848 OR902849
**c40**		44.2	x	x	x	x	3	-	-	non-mobilizable	OR902845 OR902846 OR902847
**c41**		60	x	x	x	x	2	-	-	non-mobilizable	OR902850 OR902851
**c42**		51.7	x	x	x	x	4	-	-	non-mobilizable	OR902852 OR902853 OR902854 OR902855
**c43**		63.6	*TEM-116, cfrA*	*cfr, bla*TEM-116	x	ColRNAI	1	rep_cluster_2350	-	non-mobilizable	OR902856
**c44**		46.2	*TEM-116, cfrA*	*bla*TEM-116, *cfr*	x	ColRNAI	3	rep_cluster_2350	-	non-mobilizable	OR902857 OR902858 OR902859
**c45**		58.3	x	x	x	x	1	-	-	non-mobilizable	OR902860
**a58**		66	x	x	x	Col(MGD2), Col(Ye4449), ColRNAI	2	Col(Ye4449), rep_cluster_2335	MOBP	mobilizable	OR902861 OR902862
**a60**		33.6	x	x	x	IncFII(Yp), IncFIB(K), Col(Ye4449), Col(MGD2)	4	Col(Ye4449), IncFIB, rep_cluster_2272, rep_cluster_2335	MOBF,MOBP	conjugative	OR902863 OR902864 OR902865 OR902866

Transconjugant a60 carried multi-replicon plasmid similar in identity to IncFII (Accession: CP000670, 97.83%) and IncFIB (Accession: JN233704, 97.51%), with a coverage of 98.26 and 99.29%, respectively. No ARGs were identified using CARD or resfinder, although an ampicillin-resistant phenotype was observed. Both t17 and a60 carried conjugation machinery, MOBF and MOBP suggesting that the plasmids are self-mobilizable.

The ColRNAI (Accession: DQ298019) was identified in transconjugants c36 (98.46% coverage, 85.50% identity), c43 (99.23% coverage, 86.26% identity), c44 (99.23% coverage, 86.26% identity), a58 (99.23% coverage, 83.21% identity) and a60 (99.23% coverage, 83.21% identity). The ARGs *cfrA* and *bla*_TEM-116_ were present in c36, c43 and c44. None of these plasmids conferred resistance to chloramphenicol, despite the presence of the phenicol-resistance gene *cfrA*. Only c44 was ampicillin-resistant and contained the *bla*_TEM-116_ gene, c36 had reduced susceptibility to ampicillin, whilst c42 was susceptible. For c37 and c45, we identified linear contigs while circularisation of plasmids was possible for c40, c41 and c42. However, we could not match any of the linear or circular contigs to known plasmids in the database therefore these potential plasmids require further study to identify and characterise them.

## Discussion

The presence of ARB and ARGs in drinking water is well documented across the globe. However, this has not been incorporated in surveillance systems for drinking water to-date, which leads to the lack of standards on the acceptable levels for consuming AMR bacteria or genes. In Ireland, very few studies have analysed bacteria in drinking water as a reservoir of antimicrobial resistance. Up until recently, studies that have assessed AMR in Irish supplies focused on *E. coli* and *P. aeruginosa*^20^. A more recent publication has evaluated coliforms collected by Public Health Laboratories during routine water quality testing and found a variety of bacterial species with AMR phenotypes^12^. However, the study focused solely on *Enterobacterales*, and mechanisms associated with ESBL and MBL phenotypes.

Our study incorporated citizen science to identify a comprehensive range of bacterial reservoirs of antimicrobial resistance in drinking water supplied privately to Irish households. We identified a broad range of AMR bacteria, including those usually assessed as indicators of faecal contamination (*Escherichia coli* and *Enterococcus* sp.), as well as potentially pathogenic bacteria that have yet to be recommended in water quality assessments^1,21,22^.

Most of our sampling cohort obtained their water through GWS (*n* = 30, 61%). The remaining households depended on personal private wells (*n* = 19, 39%). Contamination of privately sourced drinking water has been identified globally^23–25^. Our metadata suggests that many of the households in this study do not take action to mitigate potential microbial contamination. In 2020, the Irish Environmental Protection Agency reported 5% non-compliance rate for private supplies of drinking water. With use of membrane filtration technique for water quality testing (ISO 9308-1:2014 and ISO 7899-2:2000), it is possible that low level faecal contamination for many households is below the detection limit. Due to delays, the water samples received were at times processed beyond the 24 h timeline recommended. As a result, the loss of viable bacteria was inevitable. However, we focused on AMR analysis as faecal contamination has been previously positively correlated with ARGs^26,27^. Therefore we enriched our filtered water samples to increase the abundance of bacteria for AMR analysis.

In Ireland, septic tank leakages are often cited as the most common route of contamination of privately supplied drinking water^28,29^. We performed correlation analysis to determine associations between the reliance on septic tanks and the occurrence of antimicrobial resistant bacteria. We identified a moderate positive correlation (Spearman’s r_S_ value + 0.649, *α* = 0.001) suggesting that perhaps the septic tanks may have a role in introducing ARB to the drinking water source. We also wanted to assess whether the use of antimicrobials in the household would coincide with the detection of ARB in drinking water, as it could suggest that the household residents have (A) underwent treatment for a pathogen present in the water or (B) the use of antimicrobials have selected for these pathogens. We found a stronger correlation (Spearman’s r_S_ value of + 0.716, *α* = 0.002) in this instance. Since antimicrobials are not fully metabolised^30^, and a positive link exists between the reliance on septic tanks and the presence of ARB, it is possible that households that underwent antimicrobial treatments have selected for ARB through excrements which may have contaminated the drinking water source via septic tanks.

Our work corroborates a previous report of high prevalence of amoxicillin and amoxicillin-clavulanate resistance in *Enterobacterales* of Irish drinking water^12^ as most of our isolates were known natural producers of AmpC (e.g. *Enterobacter* sp., *Klebsiella* sp., *Citrobacter* sp.). In contrast, Daly et al., identified acquired AmpC production in *E. coli* which we did not identify in this work. While *E. coli* were isolated from four households (*n* = 40 *E. coli*), AMR *E. coli* (*n* = 25) were identified in two of the households. A total of 16 AMR *E. coli* exhibited a MDR phenotype. All of which were isolated from Household 36. The AMR phenotypes corroborated findings in another study, which identified high prevalence of ampicillin and tetracycline resistance in *E. coli* from private drinking water in Ireland^20^. In addition to this, *E. coli* isolates in our work also showed high prevalence of chloramphenicol resistance (*n* = 16, 40%) and aminoglycoside-resistance (*n* = 24, 60%). Phenicols and aminoglycosides make up a small percentage of veterinary sales in Ireland (3.3 and 7.3%, respectively)^31^. However, amongst the antimicrobials prescribed to humans, phenicols and aminoglycosides are grouped alongside the least prescribed antimicrobials, which collectively make up <10% of consumption^32^. Non-MDR *E. coli* were isolated from the two households that reported the presence of horses, sheep and cattle on or near their residence (Household 6 and Household 44), although AMR *E. coli* was only identified in one of these households (Household 6, cattle & sheep on farm) were an *E. coli* strain exhibited resistance to kanamycin. The antimicrobial resistance in our isolates echoes patterns found in clinical isolates. *Escherichia coli* is the primary cause of urinary tract (UTI) and bloodstream infection (BSI) across the EU/EEA. The majority of UTI *E. coli* isolates have been reported as resistant to at least one antimicrobial class (54.0%, 53.1%)^33,34^. High levels of AMR *E. coli* have been reported in isolates from human blood samples between 2017-2021, especially against aminopenicillins (53.1–58.7%)^34^. Cyprus, Ireland, and Bulgaria are amongst the most recurrent reporters of high levels of resistance to aminopenicillins (>60%) (https://atlas.ecdc.europa.eu/). On a global level multidrug-resistant *E. coli* have been reported in drinking water sources in Northern Tanzania and Peru^35,36^. Similar to our work, both studies report the co-existence of ampicillin and tetracycline resistance in MDR *E. coli*. While these three countries differ in terms of climate, socio-economic status, culture, antimicrobial prescribing regulations and agriculture, a commonality of the presence of these MDR *E. coli* in drinking water exists. This suggests that something else is common to all three countries. However, what this common factor is still needs to be determined. The household from which we isolated MDR *E. coli* with resistances against ampicillin and tetracycline have indicated living within 1 km from farmland. This may explain the occurrence of ampicillin and tetracycline-resistance in the isolates as penicillins and tetracycline antimicrobials made up the majority of antimicrobial sales in the veterinary industry in Ireland, making up 26.3% and 35.8% of sales in 2022^31^.

The presence of extended-spectrum β-lactamases (ESBLs) in *Enterobacterales* has been well documented in a variety of settings, including the natural environment and hospitals^37,38^. ESBL-producing *Enterobacterales* have been previously reported in drinking water in Ethiopia (*K. pneumonia*), Bangladesh (*E. coli*), and the United States (*E. coli, Klebsiella* sp*., Citrobacter* sp.)^39–41^. We did not identify ESBL phenotypes in any of our isolates. As the only species demonstrating cephalosporin-resistance in our study were known AmpC producers, AmpC over-production may have been responsible for the cephalosporin-resistant phenotypes observed^42^. In addition, the only previous study to test for ESBL-production in Irish drinking water isolates also failed to detect any ESBL phenotypes, corroborating our finding^12^.

The emergence of carbapenem-resistance amongst Gram-negative pathogens in clinical isolates has seen a rise on a global-scale^43^. Carbapenemase-producing species such as those that produce metallo-β-lactamases (MBLs) are of most concern due to the potential for horizontal gene transfer of resistance genes to pathogenic species^44^. This is significant as carbapenem antimicrobials are often used as a last-resort treatment option for MDR infections^45^. We identified carbapenem-resistance in *Stenotrophomonas maltophilia*, an emerging pathogen of concern due to its intrinsic resistance to a wide range of antimicrobial classes, including carbapenems through a chromosomally encoded MBL called L1^46^. Although considered an environmental microorganism that thrives in aquatic settings^47^, *S. maltophilia* has been identified in hospital water sources such as handwash sinks and showers^48–50^. This is likely due to both their intrinsic resistance to a broad-range of antimicrobials as well as their ability to form biofilms allowing for long term colonisation of pipes and drains^51^. This may also explain the identification of *S. maltophilia* in our household samples, as they may have colonised the water piping systems for these households. Few reports of *S. maltophilia* in drinking water are available^52,53^. The Irish EPA reports that wells are generally 60–120 m deep (https://www.epa.ie/), however five of the six households in which *S. maltophilia* was isolated in our study reported wells of depths 30 m or less. The sixth household relied on spring water. The shallow depth of wells and the exposure of springs to the natural environment may have provided an opportunity for *S. maltophilia* to enter into drinking water supplies as this species are also inhabitants of soils and rivers^54,55^.

We identified *Hafnia alvei* as a reservoir for acquired carbapenem-resistance demonstrating ertapenem-resistant phenotype. In Europe, carbapenem-resistance has mostly been associated with clinical isolates of *K. pneumonia, P. aeruginosa* and *Acinetobacter* sp.^56^ We were unable to elucidate the mechanism of carbapenem-resistance in our *H. alvei* isolate. No previous reports of aquatic carbapenem-resistant *H. alvei* exist. *H. alvei* is generally isolated from faeces of humans, animals and birds^57^ therefore its presence in drinking water could be a result of contamination by septic tank leakages or agricultural practices such as farming or manure spreading. However, the ertapenem-resistant phenotype is unusual, as the carbapenem of choice in Irish hospitals is meropenem (https://www.hpsc.ie/) which is rarely used. However, the ertapenem-resistant *H. alvei* was identified from a household that reported living in close proximity to a quarry which perhaps may be leaching heavy metals which may have co-selected for this phenotype. An OXA-48 producing *H. alvei* isolate has been reported in a clinical isolate exhibiting an ertapenem-resistance phenotype^58^. Our results suggest that the patient with the carbapenem resistant *H. alvei* may have been exposed to it from a drinking water source. This is an important finding as in recent years, infections due to this microorganism have increased predominantly in intra-abdominal focus and in immunosuppressed patients^59^.

The prescription of quinolone antimicrobials to treat human infections has reduced in the European Union as a result of a 2019 review which identified rare but serious side effects^60^. However, findings of bacteria demonstrating reduced susceptibility to the quinolones have already been established in the aquatic environments and clinical settings^61,62^. Plasmid-mediated quinolone resistance (PMQR) has been reported as early as the 1990s associated with the emergence of *qnr*, *qep* and *aac(6’)-Ib* genes^63–65^. Alternatively, mutations in the quinolone-resistance determining-regions (QRDRs) such as GyrA, GyrB and ParC can result in reduced susceptibility or resistance to the quinolones^66^. Plasmid-associated resistances against quinolone antibiotics have previously been reported in drinking water in Portugal^67^ and in other aquatic environments^68–70^. We did not identify any PMQR genes in the tested isolates. We could not elucidate the mechanism of reduced susceptibility to ciprofloxacin for some isolates of *Enterobacter* sp., *C. gillenii, S. fonticola* and *H. alvei*. However, we identified mutations mainly in the QRDRs of isolates exhibiting intermediate susceptibilities towards ciprofloxacin. The most common GyrA mutations in *E. coli* are known to occur in positions Ser-83 and Asn-87^71,72^. We identified a mutation at Asn-87 in an *E. coli* isolate. Mutations in the *gyrB* gene have mostly been reported in *Mycobacterium tuberculosis* isolated from hospital patients^73,74^ but have also been identified in *Salmonella* sp. from stool samples^75^. We identified mutations in the GyrB region of *C. gillenii* isolate 87. All mutations (positions 239, 240, 287 and 298) identified are previously unreported GyrB mutations. We also identified mutations in the GyrB region of *S. rubidaea* which may explain the reduced susceptibility in our isolates, although there are currently no other reports of GyrB mutations in *Serratia* sp. Mutations in positions 59 and 60 of the ParC region have previously been reported in clinical isolates of *Serratia marcescens*^76^. A mutation in position 57 from a clinical *E. coli* isolate (Accession: ABLAPL010000001) is reported in the NCBI Pathogen Detection Reference Gene Catalogue. Our *S. rubidaea* isolates had mutations in position 59. No previous reports of ParC mutations in *S. rubidaea* nor *H. alvei* were available. This may be because *S. rubidaea* and *H. alvei* are not considered common causes of infections and therefore have not been previously screened for QRDR mutations.

Occurrence of resistance against last-resort oxazolidinone antimicrobial linezolid has been reported in species of *Staphylococcus* and *Enterococcus*^77,78^. Linezolid is often used as a last-line defence against methicillin-resistant *Staphylococcus aureus* and vancomycin-resistant *Enterococcus* species^79^. The EARS-Net reported vancomycin-resistance in 15-18.3% of *E. faecium* between 2017 to 2021. Ireland has one of the highest prevalence of vancomycin-resistant *E. faecium* in Western Europe since 2008 (https://atlas.ecdc.europa.eu/). The only instance of VRE identified in our study was that in *E. casseliflavus* which exhibit low-level resistance against vancomycin due to a chromosomal *vanC*^80^. Linezolid-resistance in enterococci is associated with mutations in the 23 S rRNA or ribosomal L-proteins^81^, or by acquisition of ARGs *optrA, poxtA* or *cfr* via mobile genetic elements or plasmids^82–84^. Our study presents LRE species that are deficient in known mobile resistance genes (*optrA, poxtA, cfr*) but some of which contained chromosomal target site mutations. Linezolid-resistant *Enterococc*i were identified across three household samples, in *E. durans, E. faecium* and *E. casseliflavus*. Linezolid-resistant *Enterococcus* sp. were resistant to at least one other antimicrobial, namely tetracycline or ampicillin. Mutation G2576T in the 23 S rRNA are frequently associated with linezolid-resistance in *Enterococcus* sp.^81^, however the mutation we identified in *E. durans* was at a previously unreported position of the 23 S rRNA, position A2227G. Reports of mutations in the L-proteins in *Enterococcus* sp. are rare but mutation of the L3 region at codon V149I has been reported in *E. faecalis* isolated from swine^85^. Our study identified a mutation at codon S45G of the L3 protein in *E. durans*. However, contribution of this mutation to linezolid resistance requires further studies. We could not elucidate potential mechanisms of linezolid-resistance in *E. casseliflavus* and *E. faecium* as no mutations were identified in their 23 S rRNA or the ribosomal L-proteins. Linezolid-resistant *Enterococcus* sp. have been reported in clinical settings and in surface water^77,86,87^. There is currently no data available on the use of linezolid in Ireland, however, considering the high prevalence of VRE in Irish hospitals relative to other European countries, it is plausible that linezolid is frequently used for treating VRE infections. In fact, the earliest report of LRE outbreaks in an Irish hospitals began in 2014^88^. This means that the potential for selection of linezolid resistant *Enterococcus* species in Ireland in general could be higher than other countries but also the potential to select for novel linezolid resistance mechanisms is also higher.

Enrichment of our water samples for AMR analysis may have been biased towards certain species. Culture-based analysis is limited to bacteria that can withstand or thrive under the specific laboratory conditions provided. This excludes a potentially large number of species that were not culturable under these conditions and therefore the results presented may not be representative of the true composition of the drinking water samples as non-cultured bacteria may have acquired ARGs or mutations. We were unable to isolate bacteria from 21 of 49 water samples due to complete loss of viability therefore alternative approaches may have been useful such as the inclusion of molecular-based methods to overcome these caveats. For example, analysis on each drinking water sample such as metagenomics^89^ would have detected the species present, irrespective of their viability or growth requirements. Metagenomics would also allow for screening of ARGs that may have been overlooked by analysing only culturable and viable bacteria. However, studies using metagenomics at times report processing excessive volumes (20 L–2000 L) of drinking water in order to extract DNA^89–91^ making it difficult to do so without the necessary resources in place.

Our culture-dependent screening failed to identify the mechanism of resistance for a number of bacterial isolates that demonstrated an AMR phenotype. These included cephalosporin-resistant, quinolone-resistant and carbapenem-resistant *Enterobacterales* and linezolid-resistant *Enterococci*. In all of these cases, the screening involved isolating bacteria and amplifying known genes of interest via conventional polymerase chain reaction. The potential for identifying novel genes or mutations is overlooked using PCR. An alternative approach would be to perform genome sequencing of the individual isolates and analyse the entire genome for potential mutations or genes that could have contributed to the phenotypes observed^92^.

As many bacteria in drinking water tend to be viable but nonculturable^93^, we used the exogenous isolation method to capture potential mobile resistance elements. We captured plasmids within the frequently identified replicon type IncF group^94^. IncF plasmids have played a pivotal role in the successes of extraintestinal pathogenic *E. coli* (ExPEC) such as ST131 and ST410^95^. The IncF group of plasmids have previously been reported in drinking water in France and Tanzania^35,96^. They generally carried *tetA*, *bla*_TEM-1_ and *bla*_CTX-M_ genes. Our IncF-type multi-replicon plasmid t17 also contained the *bla*_TEM-1_ but carried the tetracycline-resistance *tetB* rather than *tetA* gene alongside other ARGs associated with phenicol, aminoglycoside and sulfonamide resistance. Plasmid t17 also carried virulence genes associated with iron acquisition: *iroBCDEN*, *iucABC* and*, iutA*. The *iroBCDEN* cluster originated in the chromosome of *Salmonella enterica* but has later been found on transmissible plasmids in uropathogenic *E. coli*^97^. A comprehensive study of extraintestinal pathogenic *E. coli* from veal calves found correlation between the IncFIB replicon and the presence of *iucABC*-*iutA*^98^. This supports our plasmid analysis of the multi-replicon t17 which had the IncFIB plasmid. However, this was not the case for plasmid a60 which did not carry any virulence factor genes but contained the IncFIB replicon.

The most common replicon identified in our work were the Col-type plasmids. Col-plasmids encode bacteriocin proteins called colicins, which target and kill related strains of *E. coli*^99^. This provides a colonisation advantage for the plasmid-carrying *E. coli* over related *E. coli*. The ColRNAI plasmid has been previously reported in water environments^100–102^. Other Col-plasmids identified included Col(Ye4449) and Col(MGD2). Col(Ye4449) is rarely reported but has been identified in hospital wastewater^103^ and associated with animals intended for human consumption^104,105^. Col(MGD2) has been associated with clinical and environmental settings^106–108^.

None of the ColRNAI plasmids c36, c43 and c44 conferred resistance to chloramphenicol, despite the presence of the phenicol-resistance gene *cfrA*. However, chloramphenicol-resistance in *E. coli* has frequently been associated with *cmlA, floR* and *catA* genes^109,110^ rather than *cfr*. This may explain the resistance phenotype observed in t17 which carried both *floR* and *catA* genes. As the *cfr* gene was identified on plasmids exogenously introduced into *E. coli*, the gene may have conferred AMR in its original host. The *cfr* genes are associated with phenicol and oxazolidinone-resistance amongst others in Gram-positive bacteria such as *Staphylococcus* and *Enterococcus*^111,112^. Instances of gene presence in the absence of resistance phenotypes in our transconjugants highlights the necessity of combining both phenotypic and molecular analysis for a more representative overview of the composition of AMR in drinking water. In addition, the purpose of using the exogenous method was to identify mobile resistance in a sample without relying on the cultivability of the host strain, yet mobile resistance was only found in three household samples despite the abundance of AMR bacteria in a number of household samples, some of which were multidrug-resistant. It is likely that the use of only one recipient, *E. coli* for capturing plasmids may have led to under-representation of mobile resistance as some plasmids have a narrow-host range^113^. It would have been beneficial to include an additional Gram-negative recipient such as *Klebsiella pneumonia* and Gram-positive recipients such as *Enterococcus faecium* and *Staphylococcus aureus* to reduce bias towards *E. coli*-compatible plasmids. This bias is evident in our work, as multiple types of colicin-plasmids (MGD2, Ye4449, RNAI) usually associated with *E. coli*^99^ were identified.

Annual reports on the quality of privately supplied drinking water show that Irish private supplies generally have a high rate of compliance (>95%) in respect to the absence of *E. coli*^28,29,114^. Our identification of clinically relevant and ARB in drinking water highlights the need for more robust water quality testing and surveillance. This is to minimise the risk of infection and disease in the consumers and to prolong the use of currently available antimicrobials. The results presented suggest that private Irish drinking water is a route of spread and persistence of AMR and ARG.

Overall, we demonstrate that AMR persistence and spread extends beyond the clinical setting. We have identified private household drinking water in Ireland as reservoirs for clinically relevant, antimicrobial resistant and potentially pathogenic bacteria. The detection of MDR bacteria and bacteria resistant to last-resort antibiotics was of particular concern. The transferability of AMR and virulence genes should be considered in relation to surveillance and quality testing as current water quality guidelines do not recognise ARGs as contaminants, whilst surveillance data is scarce. Surveillance of AMR may inform the transmission of mobile AMR to improve public health measures in cases of outbreaks and to subsequently preserve antimicrobials. Further studies are required to assess the pathogenicity and risk factors of the ARB identified, as drinking water is a direct link between humans and the environment in the One Health framework. This is especially important for MDR bacteria which likely carry conjugative plasmids that may support the survival of pathogens against antimicrobials.

## Methods

### Citizen science: water sampling

Households supplied with drinking water through personal private wells (*n* = 19, 39%) or Group Water Schemes (GWS) (*n* = 30, 61%) were invited to participate in this study. Participants were recruited on a voluntary basis, some of which were enroled through the National Federation of Group Water Schemes. The participating households were located in the East and Midlands of Ireland (*n* = 33, 67%) or in the West of Ireland (*n* = 16, 33%). A Group Water Scheme (GWS) is defined as any private, community-based scheme that manages and distributes drinking water to private households.

The citizens were provided with nitrile gloves and a sterile 50 mL tube. As the focus of this study was on consumed AMR, the volunteers were asked to collect water only from the tap they use for drinking water. The participants were requested to use any household disinfectant spray or wipes to sanitise the handles on their drinking water tap and countertops. The volunteers were required to fully open the tap and allow to run for 2–3 min before reducing the flow of the water to fill the tube provided. The volunteers were instructed to avoid touching the tube against the nozzle. The samples were then transported by courier to our laboratory. The participants were invited to complete a questionnaire (Supplementary Questionnaire 1) regarding the location of residence and antibiotic use in the household. They were given the option to opt-out of answering all or any of the questions.

### Isolation and identification of antimicrobial resistant bacteria from private household drinking water

Water samples (50 mL) were processed using the membrane filtration method, which is an ISO method^115^. Bacteria were subsequently enriched from membranes in Brain-Heart Infusion (Oxoid) broth at 37 °C with shaking at 225 rpm for 18 to 48 h. To select for resistant bacteria, enrichment broths were cultured on Eosin Methylene Blue (Sigma) and Slanetz & Bartley Medium agars (Oxoid) in the presence of antibiotics at breakpoint concentrations: amoxicillin (32 µg/mL) (Sigma), tetracycline (16 µg/mL) (Sigma) and ciprofloxacin (1 µg/mL) (Fluka). Bacteria were also selected in the absence of antibiotics.

Bacteria were speciated using matrix-assisted laser desorption/ionization-time of flight mass spectrometry (MALDI-TOF MS) as previously described^116,117^ using a microflex LT MALDI-TOF mass spectrometer (Bruker Daltonics) and the associated flexControl software (ver. 3.4). Spectra were classified using the Bruker Taxonomy main spectra database (MBT Compass ver. 4.1, 9607 spectra). Bacterial identification was reported to the species level if the score value was ≥ 2.00.

Any bacteria with a score between 1.70–1.99 can only be reliably identified at the genus level^118^. For each isolate with a MALDI-ToF MS score of 1.70–1.99 a single colony was suspended in 50 µL sterile deionised water and boiled in the thermocycler at 95° C for 10 min to release DNA. A PCR reaction was performed in 50 µL volumes consisting of 2.5 µL of boiled bacterial suspension, 25 µL Readymix RedTaq X2 (Sigma-Aldrich), 1 µL each of forward primer (5′-TCGTCGGCAGCGTCAGATGTGTATAAGAGACAGCCTACGGGNGGCWGCAG-3′)^119^ and reverse primers (5′-GTCTCGTGGGCTCGGAGATGTGTATAAGAGACAGGACTACHVGGGTATCTAATCC-3’)^119^ at 0.2 µM concentrations. The remainder volume was made up with sterilised deionised water. Thermocycling conditions used were as follows: initial denaturation (95 °C, 5 min), 2 cycles of denaturation (95 °C, 40 s), annealing (55 °C, 2 min), and extension (72 °C, 1 min). And a final extension (72 °C, 7 min) step. The resulting PCR products were cleaned up using AxyPrep Mag PCR Clean-up beads (Axygen) and were sent to Eurofins Genomics for Sanger sequencing. The 16 S rRNA PCR product sequences were analysed using NCBI BLASTN.

### Antimicrobial susceptibility testing using disk diffusion and broth microdilution

Antimicrobial susceptibility testing (AST) was determined by the Kirby-Bauer disk diffusion test or broth dilution method using the Clinical and Laboratory Standards Institute (CLSI) guidelines^120^.

### Correlation analysis

To assess potential correlation between reliance on domestic wastewater systems and the presence of ARB, Spearman’s rank correlation was used to analyse the relationship for households from which bacteria were isolated (*n* = 28). For this, the absence of septic tanks (*n* = 2) was denoted “1” and the use of septic tanks (*n* = 26) was denoted “2”. Similarly, the absence of viable ARB (*n* = 5) was denoted “1” while the presence of ARB (*n* = 23 households) was denoted “2”.

Further analysis was performed to investigate if there were correlations between the use of antimicrobials to treat bacterial infections, and the isolation of species of clinical relevance in household water supplies. The species of clinical relevance included *E. coli, Pseudomonas aeruginosa, Enterobacter* sp*., Enterococcus* sp., *S. maltophilia* and *Acinetobacter* sp. Households that did not indicate whether or not they have used antimicrobials in the past 10 years have been excluded from analysis (*n* = 6). Households that did not consume antibiotics (*n* = 5 households) were denoted “1”, while those who did (*n* = 17 households) were denoted “2”. The absence of species of clinical relevance was denoted “1”, while the presence was denoted “2”. The Spearman’s correlation coefficients were then compared against Spearman’s rank correlation table of critical values^121^ to identify the level of significance (*α*), where *n* is the number of household samples and *r*_*S*_ is the absolute value of the test statistic.

### Phenotypic identification of the mechanism of antimicrobial resistant *Enterobacterales*

Screening for AmpC, Extended Spectrum β-Lactamase (ESBL) and Metallo-β-Lactamase (MBL) production in the *Enterobacterales* was performed using inhibitor-based tests as previously described^122–124^. Known AmpC-producers *Citrobacter* sp.*, Enterobacter* sp.*, Hafnia alvei, Raoultella* sp., and *Serratia* sp. were excluded from AmpC testing.

### Genotype screening for carbapenemase resistance genes

Carbapenem resistant bacteria were screened for carbapenemase resistance genes using multiplex PCR. A single colony was suspended in 50 µL sterile deionised water and boiled in the thermocycler at 95 °C for 10 min to release DNA. The primers used included *bla*VIM, *bla*KPC, *bla*OXA-40, *bla*NDM, *bla*OXA-48, *bla*OXA-23, *bla*IMI, *bla*OXA-58, *bla*GES, *bla*GIM, *bla*IMP, and *bla*OXA-51 and thermocycling conditions were followed as described^125^.

### Screening for plasmid-mediated quinolone resistance and mutations in the quinolone resistance-determining regions

Isolates of potential clinical relevance showing reduced susceptibilities to ciprofloxacin were screened for plasmid-mediated quinolone resistance (PMQR) genes *qnrA, qnrB, qnrS, qepA* and *aac(6’)-Ib-cr* and mutations in their quinolone-resistance determining regions (QRDR) *gyrA, gyrB* and *parC* genes. A single colony was suspended in 50 µL sterile deionised water and boiled in the thermocycler at 95 °C for 10 min to release DNA. Genes were amplified via PCR (primers listed in Supplementary Table 2 and Table 3) and sequenced by Sanger Sequencing. BLASTX and ClustalW against reference were used to identify mutations in amino acid sequences of the QRDR regions.

### Analysis of the molecular mechanism of resistance in linezolid-resistant *Enterococcus* sp

Linezolid-resistant *Enterococcus* sp. (LRE) were screened for mobile resistance genes *optrA, poxtA* and *cfr* using PCR. Furthermore, LRE were screened for chromosomal mutations in the 23 S rRNA, L3, L4 and L22 regions. DNA was extracted using the NucleoSpin Microbial DNA kit as per manufacturer’s protocols. Primers used to amplify all genes are listed in Supplementary Table 4. The PCR products were sequenced by Sanger sequencing and analysed by BLASTN for 23 S rRNA products and BLASTX for the L-regions against reference genomes (Supplementary Table 5).

### Exogenous plasmid isolation, extraction, and sequencing

Plasmids were exogenously isolated using biparental mating as previously described using kanamycin/rifampicin-resistant *E. coli* CV601 as recipient^126^. The transconjugants were selected on LB agar supplemented with rifampicin (100 µg/mL) (Duchefa) and amoxicillin (32 µg/mL) or tetracycline (16 µg/mL) or ciprofloxacin (0.5 µg/mL). Transconjugants were screened on CHROMagar Orientation supplemented with kanamycin (64 µg/mL) (Sigma) to further confirm selection of the recipient strain. Transconjugants were subjected to AST using the CLSI guidelines for *Enterobacterales*^120^. Plasmids were extracted from all transconjugants using the Machery-Nagel NucleoSpin plasmid isolation kit and the DNA concentration quantified using Qubit-Fluorometric Quantitation.

Plasmid sequencing was performed using Oxford Nanopore Technology (ONT) MinION with the Rapid Barcoding Sequencing Kit (SQK-RBK004). Briefly, library preparation involved attaching unique barcodes and adaptors to each sample. The flow cells were primed, and the DNA library was loaded onto the flow cell for long read sequencing. The raw fast5 files were base called and demultiplexed using ONT Guppy *v6.1.2* (https://github.com/nanoporetech/rerio) with GPU acceleration. Quality control (QC) was performed using Guppy during base calling. An additional QC filter step using Filtlong *v0.2.1* (https://github.com/rrwick/Filtlong) was performed on https://usegalaxy.eu/. To remove short read sequences, the default settings were changed to exclude contigs <1000 bp.

https://usegalaxy.eu/ was used as follows: Sequences were assembled using Unicycler *v0.5.0* (https://github.com/rrwick/Unicycler) with long reads only. Reads were visualised using Bandage *v0.8.1* (https://github.com/rrwick/Bandage). ABRicate *v1.0.1* (https://github.com/tseemann/abricate) identified ARGs, virulence factors and plasmid replicon types. For all databases, the minimum DNA identity and coverage were initially set to 80% to screen for ARGs and then a second screen was performed at 50% to identify potentially more distant homologues and account for potential database bias towards clinical samples. MOB-typer *v3.0.3* (https://github.com/phac-nml/mob-suite) at default settings screened for conjugative machinery.

### Reporting summary

Further information on research design is available in the Nature Research Reporting Summary linked to this article.

## Supplementary information

Supplementary Information

REPORTING SUMMARY

## Data Availability

GenBank Accession numbers for plasmid sequences are included in Table 3.
