# Supplementary material for: Private and well drinking water are reservoirs for antimicrobial resistant bacteria
Source: NPJ Antimicrob Resist. 2024 Mar 18;2:7. doi: 10.1038/s44259-024-00024-9 (PMC11721118; doi:10.1038/s44259-024-00024-9)
Supplement: Supplementary file 1 — Supplementary Information [file 44259_2024_24_MOESM1_ESM.docx]

**Supplementary Questionnaire 1**

# **Water Sample Questionnaire**

Please answer the questionnaire accurately and to the best of your knowledge. If in doubt, please indicate this on the relevant question. An “Additional information” section is provided at the end of the questionnaire; filling this is highly encouraged. If further clarification is required for any section of the questionnaire, please contact [retracted]. Note that all questionnaires will be anonymised to ensure data protection & compliance with GDPR. This questionnaire will be used for research purposes only.

1. **Describe the location of your home by ticking the relevant box(es).**

- On a farm
- In close proximity to a farm

(Provide approximate distance, if applicable: ____)

- In a rural community
- On a remote, rural area
- In an urban region
- Near landfill
- Near quarry
- Near a manufacturing or processing company (Indicate which: ________)
- Near a pond, river, other surface water

(Specify surface water type: ________)

1. **Does your household depend on domestic wastewater treatment systems, i.e. septic tanks?**
2. **What is the source of your water supply? (Tick the appropriate box).**

- Well
- Spring
- Other (Please specify: ____________________________________________)

1. **Is this supply shared with other households?**

- Yes
- No
- Don’t know

1. **If you answered “Well” in Q3, please tick the relevant box. Otherwise, skip to Q8.**

**“My well was…”**

- Dug
- Bored
- Drilled
- Don’t know / Not applicable

1. **When was the well-constructed? (Please provide an approximate number of years, if possible).**
2. **Please provide the approximate depth of your well.**
3. **Please provide the time, date and location of sample collection (e.g. 02-FEB-2020 / 6:40am / Dunboyne, Co. Meath).**
4. **Are there any water treatment devices being used for the faucet used to collect your samples (e.g. water filters, chlorination systems etc…)? Did you remove these prior to sample collection?**
5. **How many people reside in your household?**

1. **Are any of the residents children ≤5 years old? (Please indicate no. of children aged 5 years or less).**

- Yes (#__)
- No

1. **Are any of the residents adults over 65 years old? (Please indicate no. of adults over 65).**

- Yes (#__)
- No

1. **Have any of the household residents consumed antibiotics in the past ten years? If possible, specify the type of antibiotics used (e.g. ampicillin).**
2. **If living on a farm, have any of the farm animals been exposed to antibiotic drugs at any stage since arriving on your farm? If possible, specific the type of antibiotics used (e.g. tetracycline).**

| **Please provide any additional information which you believe is relevant to either sample collection or the questionnaire. This may include any issues or deviations from sampling protocols.**  ________________________________________________________________________________________________________________________________________________________________________________________________________________________________________________________________________________________________________________________________________________________________________________________________________________________________________________________________________________________________________________________________________________________________________________________________________ |
| --- |

**Supplementary Table 1:** Date of collection vs time of processing of water samples. Bacteria were successfully isolated from all but one water samples arriving within the 24-hour window.

| **Household** | **Date of Collection** | **Date of Processing** | **Time (Hours)** | **Location** | **Culturable bacteria isolated?** |
| --- | --- | --- | --- | --- | --- |
| 1 | 11/03/2020 | 11/03/2020 | <24 | Co. Meath | Y |
| 2 | 05/10/2020 | 05/10/2020 | <24 | Co. Kildare | Y |
| 3 | 21/10/2020 | 21/10/2020 | <24 | Co. Meath | Y |
| 4 | 21/10/2020 | 21/10/2020 | <24 | Co. Meath | Y |
| 5 | 27/10/2020 | 28/10/2020 | <24 | Co. Meath | Y |
| 6 | 30/11/2020 | 01/12/2020 | <24 | Co. Wicklow | Y |
| 7 | 07/12/2020 | 08/12/2020 | <24 | Co. Meath | Y |
| 8 | 17/01/2021 | 18/01/2021 | <24 | Co. Kildare | Y |
| 9 | 24/01/2021 | 25/01/2021 | <24 | Co. Meath | Y |
| 10 | 24/01/2021 | 25/01/2021 | <24 | Co. Meath | Y |
| 11 | 31/01/2021 | 01/02/2021 | <24 | Co. Meath | Y |
| 12 | 07/02/2021 | 08/02/2021 | <24 | Co. Meath | Y |
| 13 | 07/02/2021 | 08/02/2021 | <24 | Co. Cavan | Y |
| 14 | 21/02/2021 | 22/02/2021 | <24 | Co. Meath | Y |
| 15 | 28/02/2021 | 03/03/2021 | >48 | Co. Galway | Y |
| 16 | 28/03/2021 | 30/03/2021 | 24 - 48 | Co. Laois | N |
| 17 | 28/03/2021 | 30/03/2021 | 24 - 48 | Co. Laois | Y |
| 18 | 28/03/2021 | 30/03/2021 | 24 - 48 | Co. Laois | N |
| 19 | 28/03/2021 | 30/03/2021 | 24 - 48 | Co. Laois | N |
| 20 | 28/03/2021 | 30/03/2021 | 24 - 48 | Co. Laois | N |
| 21 | 28/03/2021 | 30/03/2021 | 24 - 48 | Co. Carlow | N |
| 22 | 28/03/2021 | 30/03/2021 | 24 - 48 | Co. Carlow | N |
| 23 | 28/03/2021 | 30/03/2021 | 24 - 48 | Co. Carlow | N |
| 24 | 28/03/2021 | 30/03/2021 | 24 - 48 | Co. Carlow | N |
| 25 | 28/03/2021 | 30/03/2021 | 24 - 48 | Co. Carlow | N |
| 26 | 13/04/2021 | 16/04/2021 | >48 | Co. Galway | N |
| 27 | 13/04/2021 | 16/04/2021 | >48 | Co. Galway | N |
| 28 | 13/04/2021 | 16/04/2021 | >48 | Co. Galway | N |
| 29 | 13/04/2021 | 16/04/2021 | >48 | Co. Galway | N |
| 30 | 13/04/2021 | 16/04/2021 | >48 | Co. Galway | N |
| 31 | 13/04/2021 | 16/04/2021 | >48 | Co. Galway | N |
| 32 | 13/04/2021 | 16/04/2021 | >48 | Co. Galway | N |
| 33 | 13/04/2021 | 16/04/2021 | >48 | Co. Galway | N |
| 34 | 13/04/2021 | 16/04/2021 | >48 | Co. Galway | N |
| 35 | 13/04/2021 | 16/04/2021 | >48 | Co. Galway | N |
| 36 | 18/05/2021 | 19/05/2021 | <24 | Co. Kilkenny | Y |
| 37 | 31/05/2021 | 02/06/2021 | 24 - 48 | Co. Kilkenny | Y |
| 38 | 31/05/2021 | 02/06/2021 | 24 - 48 | Co. Kilkenny | Y |
| 39 | 31/05/2021 | 02/06/2021 | 24 - 48 | Co. Kilkenny | Y |
| 40 | 31/05/2021 | 02/06/2021 | 24 - 48 | Co. Kilkenny | Y |
| 41 | 31/05/2021 | 02/06/2021 | 24 - 48 | Co. Kilkenny | Y |
| 42 | 06/07/2021 | 09/07/2021 | >48 | Co. Clare | N |
| 43 | 06/07/2021 | 06/07/2021 | <24 | Co. Westmeath | Y |
| 44 | 06/07/2021 | 06/07/2021 | <24 | Co. Offaly | Y |
| 45 | 19/07/2021 | 20/07/2021 | <24 | Co. Galway | Y |
| 46 | 19/07/2021 | 20/07/2021 | <24 | Co. Galway | N |
| 47 | 19/07/2021 | 20/07/2021 | <24 | Co. Galway | Y |
| 48 | 19/07/2021 | 20/07/2021 | <24 | Co. Galway | Y |
| 49 | 19/07/2021 | 20/07/2021 | <24 | Co. Galway | Y |

**Supplementary Table 2:** Primers for quinolone-resistance determining regions.

| Bacterial species | Gene target | Primers (5’ – 3’) | PCR product (bp) | Annealing temp (°C) | Reference |
| --- | --- | --- | --- | --- | --- |
| *A. baumannii* | *gyrA* | F) AAATCTGCCCGTGTCGTTGGT | 344 | 58 | ^1^ |
|  |  | R) GCCATACCTACGGCGATACC |  |  |  |
|  | *gyrB* | F) GAGTGCATTAGATATTGCA | 204 | 47 | This work |
|  |  | R) GAGATCATTTTATCAAAGCG |  |  |  |
|  | *parC* | F) TTAAGTTGTCCTTGCCATTCA | 300 | 58 | ^1^ |
|  |  | R) ATGAGCGAGCTAGGCTTAAA |  |  |  |
| *Enterobacterales (Enterobacter sp., E. coli)* | *gyrA* | F) AAATCTGCCCGTGTCGTTGGT | 343 | 55 | ^2^ |
|  |  | R) GCCATACCTACGGCGATACC |  |  |  |
|  | *gyrB* | F) CAGACTGCCAGGAACGCGAT | 203 | 60 | ^3^ |
|  |  | R) AGCCAAGCGCGGTGATAAGC |  |  |  |
|  | *parC* | F) ATG TAC GTG ATC ATG GAC CG | 300 | 55 | ^2^ |
|  |  | R) ATT CGG TGT AAC GCA TCG CC |  |  |  |
| *C. gillenii* | *gyrA** |  |  |  |  |
|  | *gyrB* | F) CGGACTGTCAGGAACGCGA | 201 | 60 | This work |
|  |  | R) GCGAGCGCGGTAATCAGCG |  |  |  |
|  | *parC** |  |  |  |  |
| *H. alvei* | *gyrA* | F) AAATCGGCCCGTATCGTCGGG | 344 | 55 | This work |
|  |  | R) GCCATACCTACGGCGATACC |  |  |  |
|  | *gyrB* | F) AAGCGGCTCGTAAAGCACGT | 205 | 60 | This work |
|  |  | R) AGGATTTTACCTTTCAGCGGC |  |  |  |
|  | *parC* | F) ATGTACGTCATCATGGACAG | 301 | 55 | This work |
|  |  | R) ATTCGGTGTAACGCATGGCG |  |  |  |
| *S. fonticola* | *gyrA* | F) TGGGTGTTACGGCCAACC | 344 | 58 | This work |
|  |  | R) TCCCAGAAGGGCAGGTG |  |  |  |
|  | *gyrB* | F) AAGCGGCGCGTAAAGCGCGT | 205 | 60 | This work |
|  |  | R) AGGATTTTCCCTTTCAGCGGC |  |  |  |
|  | *parC* | F) ATGTACGTCATCATGGATCG | 301 | 55 | This work |
|  |  | R) TTCTGTATAACGCATC |  |  |  |
| *S. rubidaea* | *gyrA* | F) AAATCCGCCCGTGTCGTCGGC | 344 | 60 | This work |
|  |  | R) GCCATACCTACGGCGATACC |  |  |  |
|  | *gyrB* | F) GCAAGCTGGCGGACTGC | 213 | 60 | This work |
|  |  | R) AACCCAGCGCGGTGATCA |  |  |  |
|  | *parC* | F) ATGTACGTCATCATGGATCG | 300 | 55 | This work |
|  |  | R) TTCGGTATAACGCATC |  |  |  |

* Primers for Enterobacteriales were used to amplify the gyrA and parC genes in C. gillenii

**Supplementary Table 3:** Primers used for plasmid-mediated quinolone-resistance gene screening ^4^.

| **PMQR genes** |  | **Primer sequences** | **Amplicon size (bp)** |
| --- | --- | --- | --- |
| *qnrA* |  | CAGCAAGAGGATTTCTCACG | 630 |
|  |  | AATCCGGCAGCACTATTACTC | |
|  |  |  |  |
| *qnrB* |  | GGCTGTCAGTTCTATGATCG | 488 |
|  |  | GAGCAACGATGCCTGGTAG | |
|  |  |  |  |
| *qnrS* |  | GCAAGTTCATTGAACAGGGT | 428 |
|  |  | TCTAAACCGTCGAGTTCGGCG |  |
|  |  |  |  |
| *aac(6′)-Ib-cr* | | TTGGAAGCGGGGACGGAM | 260 |
|  |  | ACACGGCTGGACCATA |  |
|  |  |  |  |
| *qepA* |  | GCAGGTCCAGCAGCGGGTAG | 218 |
|  |  | CTTCCTGCCCGAGTATCGTG | |

**Supplementary Table 4:** Primers used for plasmid-mediated linezolid-resistance gene screening.

|  | Gene target | Primers | PCR product (bp) | Reference |
| --- | --- | --- | --- | --- |
| Linezolid resistance genes multiplex | *optrA* | F) TACTTGATGAACCTACTAACCA | 422 | ^5^ |
|  |  | R) CCTTGAACTACTGATTCTCGG |  |  |
|  | *cfr* | F)TGAAGTATAAAGCAGGTTGGGAGTCA | 746 |  |
|  |  | R) ACCATATAATTGACCACAAGCAGC |  |  |
|  | *poxtA* | F) AAAGCTACCCATAAAATATC | 533 |  |
|  |  | R) TCATCAAGCTGTTCGAGTTC |  |  |

**Supplementary Table 5:** Identification of mutations in the 23S rRNA and L-regions of linezolid-resistant Enterococcus sp.

| **Strain ID no.** | **Species** | **Linezolid resistance** | **Gene** | **Reference** | **Position** | **Mutation** | **Accession no. of susceptible comparator** |
| --- | --- | --- | --- | --- | --- | --- | --- |
| **62** | *E. durans* | Resistant | 23s rRNA | A | 2227 | G | NZ_CP022930 |
|  |  |  | *L3* | Ser | 45 | Gly |  |
|  |  |  | *L4* | - | - | - |  |
|  |  |  | *L22* | - | - | - |  |
| **76** | *E. casseliflavus* | Resistant | 23s rRNA | - | - | - | NC_020995 |
|  |  |  | *L3* | - | - | - |  |
|  |  |  | *L4* | - | - | - |  |
|  |  |  | *L22* | - | - | - |  |
| **452** | *E. faecium* | Resistant | 23s rRNA | - | - | - | NZ_CP038996 |
|  |  |  | *L3* | - | - | - |  |
|  |  |  | *L4* | - | - | - |  |
|  |  |  | *L22* | - | - | - |  |

1. Park, S. *et al.* Alterations of gyrA, gyrB, and parC and Activity of Efflux Pump in Fluoroquinolone-resistant Acinetobacter baumannii. *Osong Public Health Res. Perspect.* **2**, 164–170 (2011).

2. Vila, J., Ruiz, J., Goni, P., Marcos, A. & De Anta, T. J. Mutation in the gyrA gene of quinolone-resistant clinical isolates of Acinetobacter baumannii. *Antimicrob. Agents Chemother.* **39**, 1201–1203 (1995).

3. Liu, X., Boothe, D. M., Thungrat, K. & Aly, S. Mechanisms accounting for fluoroquinolone multidrug resistance Escherichia coli isolated from companion animals. *Vet. Microbiol.* **161**, 159–168 (2012).

4. Ciesielczuk, H., Hornsey, M., Choi, V., Woodford, N. & Wareham, D. W. Development and evaluation of a multiplex PCR for eight plasmid-mediated quinolone-resistance determinants. *J. Med. Microbiol.* **62**, 1823–1827 (2013).

5. Bender, J. K., Fleige, C., Klare, I. & Werner, G. Development of a multiplex-PCR to simultaneously detect acquired linezolid resistance genes cfr, optrA and poxtA in enterococci of clinical origin. *J. Microbiol. Methods* **160**, 101–103 (2019).
